# DNA-Sensing Antiviral Innate Immunity in Poxvirus Infection

**DOI:** 10.3389/fimmu.2020.01637

**Published:** 2020-08-28

**Authors:** Yue Lu, Leiliang Zhang

**Affiliations:** ^1^Department of Laboratory Medicine, The First Affiliated Hospital of Shandong First Medical University, Jinan, China; ^2^Institute of Basic Medicine, Shandong First Medical University and Shandong Academy of Medical Sciences, Jinan, China; ^3^Key Laboratory for Biotech-Drugs of National Health Commission, Jinan, China; ^4^Key Laboratory for Rare and Uncommon Diseases of Shandong Province, Jinan, China; ^5^Science and Technology Innovation Center, Shandong First Medical University, Shandong Academy of Medical Sciences, Jinan, China

**Keywords:** poxvirus, cGAS, DNA-PK, IFI16, STING

## Abstract

As pattern recognition receptors, cytosolic DNA sensors quickly induce an effective innate immune response. Poxvirus, a large DNA virus, is capable of evading the host antiviral innate immune response. In this review, we summarize the latest studies on how poxvirus is sensed by the host innate immune system and how poxvirus-encoded proteins antagonize DNA sensors. A comprehensive understanding of the interplay between poxvirus and DNA-sensing antiviral immune responses of the host will contribute to the development of new antiviral therapies and vaccines in the future.

## Introduction

Poxvirus is a double-stranded DNA (dsDNA) virus (1, 2) that replicates completely in the cytoplasm. Members of the *Poxviridae* family include variola virus (VARV), vaccinia virus (VACV), ectromelia virus (ECTV), and monkeypox virus (MPXV). VACV is a prototype member of the *Orthopoxvirus* genus of the *Poxviridae* family and has been used as a live vaccine for smallpox eradication (3). Interest in VACV persists because it is an excellent model for studying host pathogen interactions and cell biology (4). ECTV is a mouse-specific pathogen that has been used as a model to study the pathogenesis and immunobiology of *Orthopoxvirus* infection (5, 6).

The innate immune response against viruses is not only the first line of defense against viral infection but is also important for the establishment of adaptive immunity against viruses. The recognition of the viral DNA genome by DNA sensors, including cyclic GMP–AMP synthase (cGAS), DNA-dependent protein kinase (DNA-PK), and IFN-γ inducible protein 16 (IFI16), is the first step in the innate immune response (7, 8). Next, innate immune signal transduction is initiated by activating adaptor proteins, such as stimulator of interferon genes (STING), resulting in the production of a large number of defense molecules in the host, including interferons (IFNs) and pro-inflammatory cytokines and chemokines (9–11).

The evolutionary arms race between the virus and the host leads to the virus-mediated antagonism of antiviral immunity. Viruses hide their DNA from cellular sensing systems and/or inactivate sensors and downstream signal transduction pathways. These viral strategies include separation or modification of viral nucleic acids, interfering with specific post-translational modifications of pattern recognition receptors (PRRs) or their adaptors, degradation or cleavage of PRRs or adaptors, and separating or repositioning PRRs. For instance, there are a variety of proteins that inhibit the activation of the transcription factors interferon regulatory factor 3 (IRF3) and nuclear factor kappa B (NF-κB) or the Janus kinase (JAK)/signal transducer and activator of transcription (STAT) pathway (2).

Poxvirus encodes the largest number of immune antagonistic virus proteins, thereby showing the most diverse immune escape strategies (4). During infection, these immunomodulatory proteins are delivered to the cytoplasm of the host cell to combat the innate immune response (12, 13). The conserved central region of the poxvirus genome encodes the open reading frames (ORFs) essential for virus replication. The other ORFs are non-essential for viral replication in cell culture (14), with most associated with targeting the innate immune system (15, 16).

In this review, we summarize the DNA-sensing signal pathways in poxvirus-infected cells. In particular, we focus on DNA sensors (cGAS/DNA-PK/IFI16), an adaptor protein (STING), and host defense molecules (IFNs/cytokines). We also describe how poxvirus targets DNA sensors to abrogate the antiviral immune response. Understanding antiviral immunity and poxvirus-mediated antagonism mechanisms may guide the development of live attenuated vaccines and antiviral therapies.

## cGAS

In 2013, Sun et al. discovered a new DNA sensor, cGAS, which advanced our understanding of innate DNA sensing (17). cGAS, an enzyme belonging to the ancient oligoadenylate synthase (OAS) protein family (18), is a universal cytoplasmic DNA sensor upstream of STING. cGAS recognizes a large number of cytoplasmic DNA viruses (HSV-1, KSHV, and VACV) and retroviruses (HIV-1, HIV-2) (19–24). cGAS is activated upon binding to DNA, which catalyzes the production of 2'3'-cGAMP from ATP and GTP, resulting in the binding of second messenger cyclic GMP–AMP (cGAMP) to STING (17, 25–29). As an adaptor protein, STING recruits TBK1, which phosphorylates IRF3. Then, IRF3 is relocated to the nucleus to induce IFN and thus establishes an antiviral state (19, 30–33). NF-κB is also activated by STING (32).

The cGAS–STING pathway is very important for sensing ECTV infection, inducing type I IFN production and controlling ECTV replication (34). In the lymph nodes of mice infected with ECTV, inflammatory monocytes (IMOs) are the main cells producing type I IFN in draining lymph nodes (DLNs). To induce the expression of IFN and pro-inflammatory cytokines, IMOs require STING–IRF7 and STING–NF-κB (10).

By using cGAS-deficient mice, researchers showed that type I IFN is not produced during VACV infection (35, 36). In addition, cGAMP, produced by cGAS in virus-infected cells, can be transferred to uninfected neighboring cells through gap junctions, where it promotes STING activation and antiviral immunity reactions independent of type I IFN (37).

Interferon-induced oligoadenylate synthetase-like (OASL) binds specifically to cGAS and inhibits cGAS enzyme activity in the process of DNA virus infection, which inhibits IFN induction and promotes DNA virus replication through the cGAS–STING DNA sensing pathway (38). Deletion of human OASL and mouse OASL2 can inhibit DNA virus infection. OASL1 and OASL2 are negative feedback regulators of cGAS and inhibit cGAS-mediated type I IFN induction (38).

The modified VACV Ankara strain (MVA) has been designed as a vaccine vector (39–41), and it can effectively prevent VARV and MPXV infection (42, 43). IFN in MVA-infected conventional dendritic cells (cDCs) is produced independently of the RNA-sensing pathway mediated by MDA5, MAVS, TLR3, or TRIF and is not affected by the absence of TLR9/MyD88 in the DNA sensing pathway *in vivo*. The cGAS/STING-mediated DNA-sensing pathway plays a key role in MVA-induced IFN production in CDCs. MVA infection of cDCs triggers the phosphorylation of TBK1 and IRF3, which is abolished in the absence of cGAS and STING. Similar results were also observed in mouse models (44).

## TLR9

Of the 10 TLRs found in humans, TLR9 is the only known DNA sensor. TLR9 specifically recognizes the unmethylated CpG motif in dsDNA (CpG DNA), which is common in bacterial and viral genomes (32, 45–47). TLR9 recruits the adaptor protein MyD88 and then recruits tumor necrosis factor receptor associated factor 6 (TRAF6) and IκB kinase (IKK) complexes; the former leads to the activation of IRF7 and ultimately induces the production of type I IFN (48, 49), and the latter leads to the activation of NF-κB, resulting in the induction of inflammatory cytokines (50).

TLR9/MyD88 sensing increased the expression of the NKG2D ligand in virus-infected migratory dendritic cells (mDCs), and induced production of IFN-γ in classical NK cells and innate lymphoid cells (ILCs). IFN-γ induces CXCL9 in uninfected IMOs and induces the recruitment of protective NK cells to DLNs (51). In CD11c^+^ cells, MyD88–IRF7 recruit IMOs to DLNs, and although the TLR9–MyD88–IRF7 signaling pathway is necessary for IMOs recruitment to DLNs, it is not directly necessary for type I IFN production. The induction of type I IFN in DLNs during ECTV infection is due to the indirect recognition of the virus by the TLR9–MyD88–IRF7 and STING–IRF7/NF-κB pathways (52). Compared with wild-type mice, mice lacking TLR9 and MyD88 showed higher viral loads, more severe pathological liver and spleen conditions, and increased susceptibility to ECTV infection (53–55). C57BL/6 mice lacking IRF7 and NF-κB, which are downstream targets of TLR9–MyD88 and STING, are highly susceptible to ECTV infection (52).

## AIM2

Absent in melanoma 2 (AIM2), a member of the PYHIN protein family, is a receptor of cytoplasmic DNA. AIM2 senses viral DNA and can activate the inflammasome pathway (56, 57), which plays an important role in the production of pro-inflammatory cytokines and the clearance of infected cells through pyroptosis (58). After AIM2 binds to DNA through its HIN200 domain, caspase-1 is recruited and activated, leading to the production of inflammatory cytokines, including IL-1β and IL-18. Disabling AIM2 inhibits caspase-1 activation by cytoplasmic dsDNA and VACV infection (59, 60). More importantly, AIM2-deficient cells have a defective innate immune response to VACV (61).

## IFI16, DNA-PK, and Other DNA Sensors

IFI16, a member of the PYHIN protein family, recognizes the DNA virus genome in the nucleus and activates antiviral gene expression and the inflammasome-mediated immune response. IFI16 is mainly located in the nucleus but can also shuttle between the cytoplasm and nucleus in different types of cells (62). IFI16 could bind to the dsDNA fragment of 70 bp from the VACV genome (48). It can also interact with STING to induce the TBK1-dependent IFN-β response. The nuclear induction of IFI16 upon cell exposure to viral DNA activates the inflammasome pathway through ASC and caspase-1, resulting in the production of IL-1β and IL-18 (63).

Both IFI16 and cGAS are necessary for the activation of STING, which is induced by cGAMP. They interact with STING to promote its phosphorylation and translocation. IFI16 is the main nuclear DNA receptor, while cGAS plays an auxiliary role. For example, upon the stabilization of IFI16 to initiate or prolong signal enhancement, the synergistic effect of IFI16 and cGAS can induce immune signaling in response to exogenous DNA in the nucleus (64, 65).

DNA-PK is a protein kinase that binds to cytoplasmic DNA. It is composed of Ku70, Ku80, and catalytic subunit DNA-PKCs. In the case of VACV infection, DNA-PK relies on STING, TBK1, and IRF3 to induce cytokine production (32, 45–47). PRR detection of DNA triggers the production of type I IFN, cytokines, and chemokines through the STING pathway (51).

DNA viruses usually release genomic DNA into the nucleus of host cells after entry. Heterogeneous nuclear ribonucleoprotein A2B1 (HnRNPA2B1) recognizes viral DNA, undergoes homodimerization, and is demethylated by arginine demethylase JMJD6 at Arg226. This modification results in hnRNPA2B1 translocation to the cytoplasm and activation of the TBK1–IRF3 pathway, which enhances IFN-α/β production. In addition, hnRNPA2B1 promotes the modification of N6-methyladenosine (m6A) and the nuclear and cytoplasmic transport of cGAS, IFI16, and spiny mRNA. These factors mediate the amplified activation of the cytoplasmic TBK1–IRF3 pathway. Therefore, nuclear hnRNPA2B1 initiates and amplifies the innate immune response to DNA viruses (52).

RNA polymerase III is a new type of dsDNA cytoplasmic DNA sensor, and RIG-I is pivotal in sensing viral RNA. AT-rich dsDNA serves as a template for this DNA sensor, RNA polymerase III converts poly(dA:dT) to poly(A:U)-rich dsRNA, which, in turn, serves as a RIG-I agonist. Then, activation of RIG-I by this dsRNA induces the production of type I IFN and activation of the transcription factor NF-κB (53–55).

## Viral Antagonism

Poxvirus inhibits innate immunity through diverse mechanisms that involve multiple players including sensors, adaptors, and effectors. In this review, we focus on sensors and the most recent studies on adaptors and effectors. Therefore, only a small number of poxvirus immune antagonistic proteins are discussed. More poxvirus immune evasion mechanisms have been summarized in previous studies (4, 66–69).

## DNA Sensors

cGAS is the main sensor that mediates IRF activation and ISG response to VACV lacking F17 (44, 70). The poxvirus F17 protein hijacks the mammalian target of rapamycin (mTOR) regulatory factors Raptor and Rictor, leading to an mTOR imbalance. Excess mTOR accumulates in the Golgi apparatus and causes mTOR-dependent cGAS degradation, thus inactivating the cGAS–STING pathway (71). In contrast, when VACV lacking F17 infects the cells, cGAS activates STING. Then, STING is phosphorylated, dimerized, and translocated from the endoplasmic reticulum (ER) to the perinuclear region, where it mediates the activation of IRF3 (72).

DNA-PK can be antagonized by VACV proteins C16 and C4. C16 and C4 bind to Ku and block the binding of Ku to DNA (73), resulting in the reduced production of cytokines and chemokines, decreased recruitment of inflammatory cells, and inhibition of IRF3 signaling. The response to VACV infection is weakened in cells and mice lacking DNA-PK components (49). A model infected with C16-knockout VACV show fewer signs of disease and upregulated cytokine synthesis (73, 74). C4 inhibits NF-κB signaling (75) and cytokine production *in vitro* and *in vivo*. The loss of C4 enhances the recruitment and activation of cells involved in innate and acquired immunity.

## Adaptors

Georgana et al. studied the activation of innate immune signals by four different VACV prototypes. They found that the virulent Copenhagen and Western Reserve VACV strains inhibited STING dimerization and phosphorylation during infection and in response to transfected DNA and cGAMP, thus effectively inhibiting DNA sensing and the activation of IRF3. However, an attenuated MVA strain showed the opposite result, and IRF3 was activated by cGAS and STING after infection (70). Georgana et al. found that virus-encoded protein C16 is a viral DNA sensing inhibitor that acts upstream of STING and has the ability to block STING activation (70).

## Downstream Signaling Molecules

The mutation of serine to alanine in the IκBα-like motif of A49 prevented β-TrCP binding, stabilized p-IκBα and inhibited the activation of NF-κB (76). B14 targets IKK complex and inhibits the activation of NF-κB in response to TNF-α, IL-1β, Poly(I:C), and PMA (77). The intracellular immunomodulatory proteins K1L, N1L, and A52R can inhibit the NF-κB signaling pathway (44, 78, 79).

VACV virulence factor N1 is a 14 kDa cytoplasmic protein that facilitates an increase in virulence (80, 81) and plays an inhibitory role in the cGAS–STING–IRF3-dependent cytoplasmic DNA-sensing pathway and in IFN-β gene induction (82).

Poxvirus protein serine protease inhibitor 2 (SPI-2) and cytokine response modifier (CrmA) are involved in a variety of poxvirus immune escape strategies. SPI-2 and CrmA target caspase-1 to prevent apoptosis and cytokine activation. The ectopic expression of SPI-2 or CrmA inhibits the induction of IFN-β and its downstream genes. SPI-2 and CrmA can also bind to TBK1 and IKKε to disrupt the STING-TBK1/IKK ε-IRF3 complex, which is a newly discovered mechanism of the SPI-2/CrmA–mediated immune escape of poxvirus (83).

VACV expresses many proteins that antagonize the IFN system. C6 is a multifunctional IFN inhibitor expressed prior to viral genome replication and resides in the cytoplasm and nucleus. It can reduce IFN production and inhibit IFN-induced signal transduction, thus inhibiting ISG expression (84). C6 inhibits the activation of IRF3 by binding to TBK1 in the cytoplasm, thus blocking the induction of IFN by IRF3 (84).

Poxvirus encodes several soluble IFN receptors. For instance, VACV B8 interacts with IFN-γ and prevents it from binding to IFN-γ receptors (85–87). VACV B18 binds to type I IFN and blocks the signal transduction of IFNAR (88–91).

## Conclusion and Perspectives

**Figure 1:**
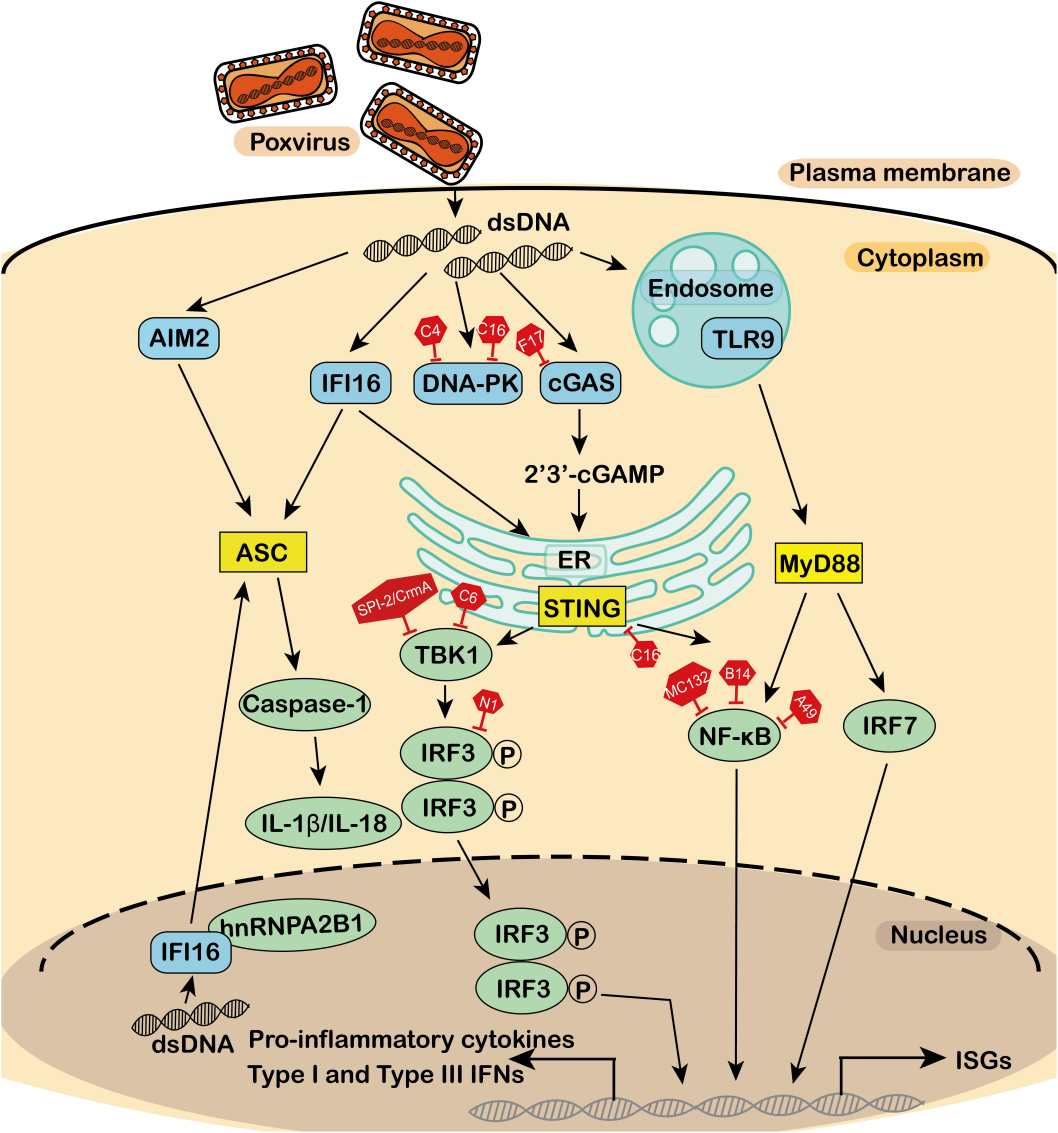
Antagonism of the DNA sensor by poxvirus. During poxvirus infection, the cytosolic DNA sensor activates the adaptor, which in turn activates a series of downstream effectors to produce interferons, cytokines, and interleukins for an antiviral immune response. DNA sensors, adaptors, effectors, and virus-encoded inhibitors are in blue, yellow, green, and red, respectively. dsDNA, double-stranded DNA; TLR9, toll-like receptor 9; IFI16, interferon-γ inducible protein 16; cGAS, cyclic guanosine monophosphate-adenosine monophosphate synthase; DNA-PK, DNA-dependent protein kinase; AIM2, absent in melanoma 2; MyD88, myeloid differentiation factor 88; STING, stimulator of interferon genes; ASC, apoptosis-associated speck-like protein containing a CARD; IFN, interferons; CK, cytokines; NF-κB, nuclear factor κB; TBK1, TANK-binding kinase 1; IRF3, interferon regulatory factor 3; P, phosphorylation; IRF7, interferon regulatory factor 7; IL, interleukin; HnRNPA2B1, heterogeneous nuclear ribonucleoprotein A2B1; ER, endoplasmic reticulum; ISGs, IFN stimulating genes.

In this review, we discussed the interplay between poxvirus and host antiviral innate immune factors, particularly focusing on the STING pathway (Figure 1). The sensor proteins upstream of STING are cGAS, DNA-PK, and IFI16. There are two pathways of downstream STING effectors: TBK1-IRF3 and IKK-NF-κB. These two signaling pathways induce the production of IFNs and cytokines. In addition, we also described other signaling pathways that trigger the innate immune response.

Subcellular compartments are involved in the spatiotemporal interplay between poxviruses and DNA sensing molecules. TLR9 is located in endosomes, while STING is located in the ER. Yip1 Domain Family Member 5 (YIPF5) is recycled between the ER and the Golgi, involving the maintenance of the Golgi structure. YIPF5 recruits STING to COPII vesicles and facilitates STING trafficking from the ER to the Golgi apparatus, triggering type I IFN production (92). Interestingly, cGAS and IFI16 are located in the nucleus and cytoplasm. Acetylation of nuclear localization signal sequences targets IFI16 to the cytoplasm, thus fine-tuning the subcellular distribution of IFI16. Endogenous cGAS seems to be uniformly distributed in the cytoplasm and nucleus (93). Although poxvirus replicates in the cytoplasm, many viral proteins are located in the nucleus. cGAS and IFI16 are partially localized to the nucleus; however, no nuclear poxvirus proteins are reported to antagonize cGAS or IFI16 in the nucleus.

To successfully survive, the poxvirus genome encodes a number of immunomodulatory proteins to escape the innate immune response. The key challenge is to translate the viral evasion mechanism into useful applications for the development of new vaccines and antiviral drugs. Knockouts of immunomodulatory proteins or the depletion of specific viral PRR antagonistic mechanisms may lead to changes in virulence and/or the immune response, which may effectively induce long-lasting immune antiviral responses and may improve the immunogenicity of viral vectors.

Through these recent achievements, we have gained a richer understanding of viral evasion mechanisms in host cells. However, there are gaps that need to be investigated further. Firstly, how the interplay between poxvirus and innate immune response affects human viral diseases is unknown. Secondly, what are the relative contributions of the many DNA sensors required for poxvirus sensing? There is no definite answer to date. Finally, what might be the unique viral ligands that activate distinct DNA sensors? Are these DNA sensors involved in different cell types? Determining the molecular mechanism of poxvirus evasion will not only greatly contribute to important insights for the development of antiviral drugs and vaccines but will also provide a viral model for the future study of viral antagonism to host immunity.

## Author Contributions

LZ conceived the work and modified the manuscript. YL drafted the manuscript. All authors contributed to the article and approved the submitted version.

## Conflict of Interest

The authors declare that the research was conducted in the absence of any commercial or financial relationships that could be construed as a potential conflict of interest.
